# Airborne Relay-Based Regional Positioning System

**DOI:** 10.3390/s150612682

**Published:** 2015-05-28

**Authors:** Kyuman Lee, Hongjun Noh, Jaesung Lim

**Affiliations:** 1 Department of Computer Engineering, Ajou University, 206 World cup-ro, Yeongtong-gu, Suwon 443-749, Korea; E-Mail: mool717@ajou.ac.kr; 2 Communication Research Center, LIG Nex1, 333 Pangyo-ro, Bundang-gu, Seongnam 463-400, Korea; E-Mail: hongjun.noh@lignex1.com

**Keywords:** ground-based positioning, airborne relay, GNSS-independent system

## Abstract

Ground-based pseudolite systems have some limitations, such as low vertical accuracy, multipath effects and near-far problems. These problems are not significant in airborne-based pseudolite systems. However, the monitoring of pseudolite positions is required because of the mobility of the platforms on which the pseudolites are mounted, and this causes performance degradation. To address these pseudolite system limitations, we propose an airborne relay-based regional positioning system that consists of a master station, reference stations, airborne relays and a user. In the proposed system, navigation signals are generated from the reference stations located on the ground and are relayed via the airborne relays. Unlike in conventional airborne-based systems, the user in the proposed system sequentially estimates both the locations of airborne relays and his/her own position. Therefore, a delay due to monitoring does not occur, and the accuracy is not affected by the movement of airborne relays. We conducted several simulations to evaluate the performance of the proposed system. Based on the simulation results, we demonstrated that the proposed system guarantees a higher accuracy than airborne-based pseudolite systems, and it is feasible despite the existence of clock offsets among reference stations.

## 1. Introduction

The need for location information has been increasing on account of the growth of the location-based service industry. The Global Navigation Satellite System (GNSS) is used in many areas, because it can globally provide accurate positioning, navigation and timing (PNT) services. However, GNSS may not be available in shaded areas, and it is vulnerable to jamming due to weak signals. Several studies have been conducted to develop alternatives [1–4].

A positioning concept was proposed that employs multiple ground-based pseudolites, which are transmitters of GNSS-like signals, and provides navigation signals through these pseudolites [3]. This ground-based positioning approach has the advantage of a higher received signal power than GNSS, as well as greater flexibility, because pseudolites can easily be installed in any location. In addition, because all system components are on the ground, the construction and maintenance are simple and cost-effective. Therefore, various ground-based positioning and navigation applications have been studied [3–8]. Nevertheless, this ground-based positioning system can be impacted by the multipath effect; moreover, when all given pseudolites and receivers are placed in the same plane, the system may not precisely estimate the receiver altitude due to poor vertical observability [8]. Furthermore, the proportional change in distances between pseudolites and the user becomes considerably large when the pseudolites and user are close. Thus, the near-far problem occurs, because the received power level of the pseudolite signals can vary over a large range [7].

As another approach, the airborne-based pseudolite positioning system has been studied in conjunction with the ground-based system. In this system, the pseudolites are mounted on fixed-wing aircraft, helicopters, unmanned aerial vehicles (UAVs) and stratospheric airships to provide navigation signals [9–12]. Unlike the ground-based pseudolite system, the near-far problem and multipath effect are not significant, because the distances between the pseudolites and user range from 2^0^ km to 100 km, and the line-of-sight (LOS) is guaranteed. Moreover, the airborne-based system provides a larger coverage area and better vertical observability than the ground-based system [9]. However, because the platforms on which the pseudolites are mounted continuously move, the pseudolite positions must be accurately monitored in real time to provide precise navigation services [12]. The pseudolite positions are calculated using the measurements and position information of ground receivers at a ground control station. Therefore, some delay due to monitoring can occur, and changes in pseudolite position during monitoring cause performance degradation. Chandu [12] analyzed the impact of performance due to the movement and monitoring time of pseudolites.

The Chinese Area Positioning System (CAPS) was proposed [13]. It differs from other positioning systems in that its navigation signals are generated from a ground station and delivered to users through communication satellites. The user calculates the pseudoranges between the satellites and himself/herself by subtracting the uplink times between the ground station and satellites from the total navigation signal propagation times and then estimates his/her position using these pseudoranges. Therefore, the uplink times and satellite positions must be measured in advance and included in the navigation signals. Consequently, if the relay platforms, such as satellites, are mobile, this positioning concept is limited, because it is difficult to apply the position of relays in real time to estimate the user position.

In this paper, we propose a regional positioning system that employs airborne relays to resolve the limitations of pseudolite-based positioning systems. The proposed system consists of a master station, reference stations, airborne relays and a user. To perform positioning, the reference stations located on the ground generate navigation signals, and the airborne relays transfer these signals to the user. The user then sequentially estimates the airborne relay positions and his/her own position using the relayed signals. Owing to these structural and procedural characteristics, the proposed system has advantages over the pseudolite-based positioning system and CAPS. Specifically, the monitoring and measuring of information for positioning, such as the positions of airborne relays and the propagation time between reference stations and airborne relays, are not required, because the user can simultaneously estimate the airborne relay positions and his/her own position. Furthermore, the mobility of airborne relays does not affect the accuracy of the system. Thus, the proposed system provides greater flexibility in application. We conducted simulations to evaluate the performance of the proposed system. The simulation results demonstrate that the proposed system is feasible and more accurate than the airborne-based pseudolite positioning system.

The rest of this paper is organized as follows. In Section 2, the airborne-based pseudolite system and positioning algorithm are detailed. In Section 3, the proposed airborne relay-based positioning system and positioning algorithm are described. The simulation construction and results are presented in Section 4. Finally, the paper is concluded in Section 5.

## 2. Airborne-Based Pseudolite Positioning System Based on the IGPS Method

The proposed system is a hybrid structure that is configured with ground reference stations and airborne relays for regional positioning. It is similar to the airborne-based pseudolite positioning system in terms of structure and operation environments. Therefore, we compare the proposed system with the airborne system. In the airborne-based pseudolite positioning system, in which the pseudolite platforms continually move, monitoring pseudolite position is important, because the pseudolite position accuracy affects user accuracy. Two methods estimate pseudolite positions: the Inverted Global Positioning System (IGPS) method and the transceiver method. In this study, we consider the IGPS method for monitoring, because its accuracy levels are higher than the transceiver method [12].

**Figure 1:**
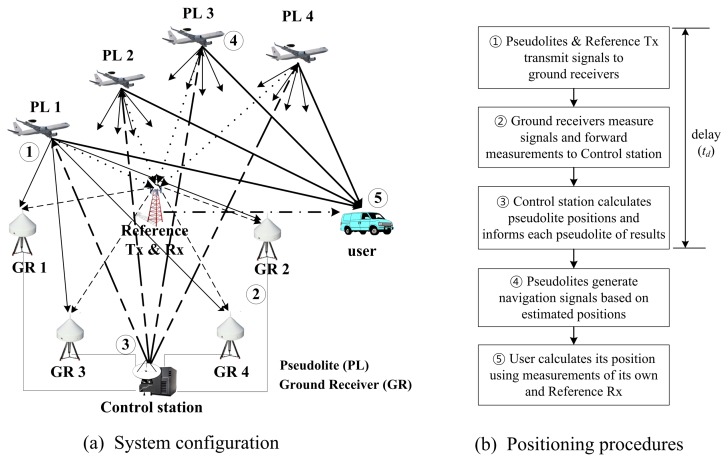
Airborne-based pseudolite positioning system based on the Inverted Global Positioning System (IGPS) method.

As shown in Figure 1a, the IGPS-based airborne pseudolite system is comprised of airborne pseudolites, ground receivers, a control station, a reference transmitter, a reference receiver and a user. The ground receivers are connected to the control station; their positions are known. The reference transmitter and receiver are required to provide double-differenced measurements for eliminating clock offsets of the ground receivers, pseudolites and user.

Figure 1b shows the positioning procedures. The pseudolites and reference transmitter transmit signals to the ground receivers. Each ground receiver measures these signals and forwards them to the control station. The control station calculates the pseudolite positions using double-differenced measurements and the known positions of the ground receivers. It then informs each pseudolite of these results [9]. Based on the estimated position, each pseudolite generates its navigation signal. The user receives these signals and calculates its position. If the pseudolites and user are asynchronized, the user performs double-differencing of the reference receiver measurements and his/her own measurements. As shown from the procedure, the pseudolite positions are estimated by the control station. Therefore, some delay occurs, and the position changes of the pseudolites during monitoring decrease the user accuracy.

For comparison, we reformulate the navigation equations of the airborne-based pseudolite system in Appendix A.

## 3. Proposed Airborne Relay-Based Regional Positioning System

### 3.1. System Configuration

**Figure 2:**
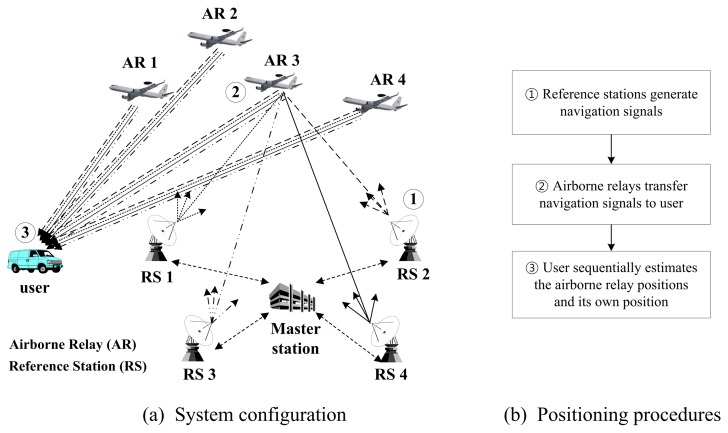
Configuration of the proposed positioning system.

In this section, the proposed airborne relay-based regional positioning system is detailed. The proposed system is comprised of a master station, reference stations, airborne relays and a user terminal. The system configuration is shown in Figure 2a.

The master station is a means for providing an absolute time reference for the system. It mounts an atomic clock and exchanges messages with reference stations for time synchronization. Furthermore, it generates information for augmentation by receiving the relayed navigation signals transferred from the airborne relays. The reference stations are transmitters that generate navigation signals based on their known positions and time data, similar to pseudolites and GNSS satellites. All reference stations should be synchronized with each other in accordance with the master station, and they should periodically broadcast navigation signals. The reference stations are basically configured at fixed positions; however, they can be designed as mobile stations if necessary. In that case, the exact positions or trajectories of the reference stations must be known. Airborne relays constitute new segments not found in the conventional ground-based positioning system. These airborne relays send navigation signals back to the user via different frequencies as soon as they are received from the reference stations, similar to bent-pipe satellites. The proposed system can achieve the advantages of both ground-based and airborne-based pseudolite positioning systems by employing airborne relays. Unlike other positioning systems, the user of the proposed system estimates both the positions of the airborne relays and his/her own position using relayed navigation signals. For this positioning, at least four reference stations and four airborne relays should be configured. The user procedure is described in more detail in the following section.

### 3.2. User Positioning Algorithm

As can be seen in Figure 2b, the positioning procedure of the proposed system is simple compared to that of the airborne-based pseudolite system. According to this procedure, the reference stations generate navigation signals, and the airborne relays transfer these signals to the user. After receiving the signals, the user performs the positioning. Unlike in CAPS, because the navigation signals do not include information of relays, such as positions and propagation times from reference stations to airborne relays, the user must precisely estimate the positions of the airborne relays before estimating his/her own position. In this paper, we proposes a two-step positioning algorithm at the user terminal. The mathematical expression of the estimation procedures is derived as follows.

**Figure 3:**
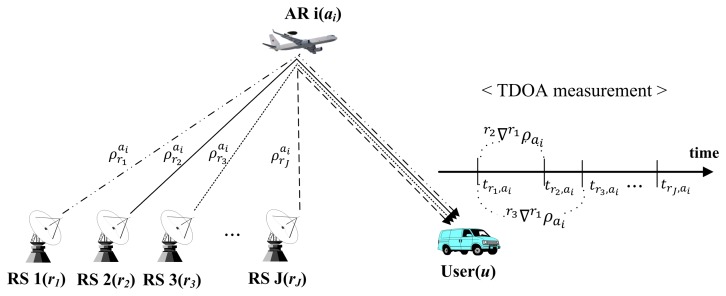
Time difference of arrival (TDOA) measurement in the first step.

The first step of the proposed positioning algorithm is the position estimation of the airborne relays. As shown in Figure 3, the user measures the time difference of arrival (TDOA) of the navigation signals simultaneously generated from each reference station and relayed by each airborne relay For *I* airborne relays and *J* reference stations, the difference of pseudoranges between the first/*j*-th reference stations and *i*-th airborne relay can be derived as:

(1) $$\begin{array}{cc} c\left(t_{r_{j},a_{i}}-t_{r_{1},a_{i}}\right)=\rho_{r_{j}}^{a_{i}}-\rho_{r_{1}}^{a_{i}}{=}^{r_{j}}\nabla^{r_{1}}\rho_{a_{i}}, & 1\leq i\leq I,1\leq j\leq J \end{array}$$

where *c* denotes the speed of light, *t*_*r_j_*,_*_a_i__* is the reception time of the relayed signal by the *i*-th airborne relay from the *j*-th reference station to the user and $\rho_{r_{j}}^{a_{i}}$ represents the pseudorange between the *i*-th airborne relay and *j*-th reference station. From Equation (1), the difference of pseudoranges with the *j*-th reference station based on the first reference station is rewritten as:

(2) ∇rjr1ρai=eairj⋅(Rrj−Rai)−eair1⋅(Rr1−Rai)−brj+br1+∇rjr1τai+∇rjr1mai+∇rjr1nai≡eairj⋅(Rrj−Rai)−eair1⋅(Rr1−Rai)−brj+br1+∇rjr1ɛai

where $e_{r_{j}}^{a_{i}}$ is the unit vector from the *i*-th airborne relay to the *j*-th reference station, ***R****^ai^* and ***R****_rj_* denote the position vectors of the *i*-th airborne relay and *j*-th reference station, respectively, *b_rj_* represents the *j*-th reference station clock offset and τ, *m* and *n* signify the tropospheric delay, multipath delay and thermal noise, respectively.

The airborne relay positions are estimated using the known positions of reference stations and TDOA measurements. Using Equation (2)*J* − 1 equations are determined in the same way for the *J* reference stations. The navigation equation for estimation of the *i*-th airborne relay is constructed as:

(3) [eair1−eair2eair1−eair3⋮eair1−eairJ]︸Hrel⋅Rai=[∇r2r1ρai−eair2⋅Rr2+eair1⋅Rr1+br2−br1−∇r2r1ɛai∇r3r1ρai−eair3⋅Rr3+eair1⋅Rr1+br3−br1−∇r3r1ɛai⋮∇rJr1ρai−eairJ⋅RrJ+eair1⋅Rr1+brJ−br1−∇rJr1ɛai]︸Prel

(4) $$H_{\text{rel}}\cdot R^{a_{i}}=P_{\text{rel}}$$

The *i*-th airborne relay position is determined by the nonlinear least squares method using the Levenberg-Marquardt algorithm [14]. The user repeats the above procedures until all airborne relay positions are estimated.

**Figure 4:**
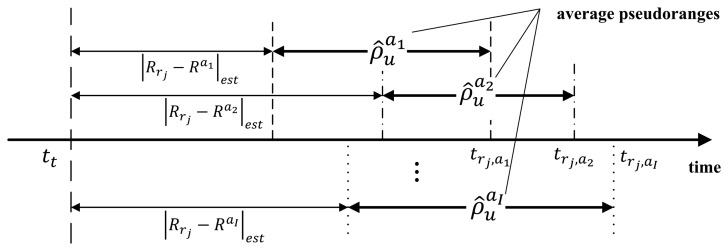
Estimation of pseudoranges between the airborne relays and user.

After position estimation of the airborne relays, the user position is determined in the second step. A navigation signal includes a time stamp, which indicates when the signal was transmitted from a reference station. As shown in Figure 4, the user calculates the pseudorange from each airborne relay to the user using these time stamps and the estimated positions of airborne relays in the first step.

In the proposed system, the user can calculate multiple pseudoranges for each airborne relay, because the airborne relay transfers all navigation signals, which are generated from all reference stations. However, each navigation signal measurement has a different error in accordance with the location of the reference stations. Thus, the calculated pseudorange can vary depending on which navigation signal is used. Consequently, the average of all pseudoranges for each relay is used to mitigate the influence by a geometrical factor; we can expect that more precise pseudoranges are estimated. The average pseudorange is represented as follows:

(5) $${\hat{\rho }}_{u}^{a_{i}}=\frac{1}{J}\sum_{k=1}^{J}\left(c\left(t_{r_{k},a_{i}}-t_{t}\right)-{\left|R_{r_{k}}-R^{a_{i}}\right|}_{\text{est}}\right)$$

where ${\hat{\rho }}_{u}^{a_{i}}$ is the average pseudorange between the *i*-th airborne relay and user, *t_t_* denotes the transmission time of the navigation signal at all reference stations and |***R****_rk_* − ***R****^ai^*|*_est_* represents the estimated distance between the *k*-th reference station and *i*-th airborne relay. The pseudorange is rewritten as:

(6) $${\hat{\rho }}_{u}^{a_{i}}=e_{u}^{a_{i}}\cdot \left(R^{a_{i}}-R_{u}\right)-{\hat{b}}_{r}+{\hat{\tau }}_{u}^{a_{i}}+{\hat{m}}_{u}^{a_{i}}+{\hat{n}}_{u}^{a_{i}}+ct_{u}$$

where *b̂_r_* denotes the average clock offset of reference stations, *τ̂*, *m̂* and *n̂* indicate the average tropospheric delay, multipath delay and thermal noise, respectively, and *t_u_* is the clock offset of user.

Equation (6) can then be expanded into the following set of equations for *I* airborne relays. Consequently, the user can obtain its position and clock offset by the nonlinear least squares method as in the first step:

(7) $$\left[\begin{array}{cccc} a_{x}^{a_{1}} & a_{y}^{a_{1}} & a_{z}^{a_{1}} & -c\\ a_{x}^{a_{2}} & a_{y}^{a_{2}} & a_{z}^{a_{2}} & -c\\ \vdots & \vdots & \vdots & \vdots \\ a_{x}^{a_{I}} & a_{y}^{a_{I}} & a_{z}^{a_{I}} & -c \end{array}\right]\;\left[\begin{array}{c} x_{u}\\ y_{u}\\ z_{u}\\ t_{u} \end{array}\right]=\left[\begin{array}{c} e_{u}^{a_{1}}\cdot R^{a_{1}}-{\hat{\rho }}_{u}^{a_{1}}-{\hat{b}}_{r}+{\hat{\tau }}_{u}^{a_{1}}+{\hat{m}}_{u}^{a_{1}}+{\hat{n}}_{u}^{a_{1}}\\ e_{u}^{a_{2}}\cdot R^{a_{2}}-{\hat{\rho }}_{u}^{a_{2}}-{\hat{b}}_{r}+{\hat{\tau }}_{u}^{a_{2}}+{\hat{m}}_{u}^{a_{2}}+{\hat{n}}_{u}^{a_{2}}\\ \vdots \\ e_{u}^{a_{I}}\cdot R^{a_{I}}-{\hat{\rho }}_{u}^{a_{I}}-{\hat{b}}_{r}+{\hat{\tau }}_{u}^{a_{I}}+{\hat{m}}_{u}^{a_{I}}+{\hat{n}}_{u}^{a_{I}} \end{array}\right]$$

where $a_{x}^{a_{i}}$, $a_{y}^{a_{i}}$ and $a_{z}^{a_{i}}$ are the LOS elements in the respective x, y and z directions from the *i*-th airborne relay to the user and *x_u_, y_u_* and *z_u_* represent the user position.

In the proposed positioning algorithm, the user can sequentially calculate the positions of airborne relays using the TDOAs of relayed signals and his/her position based on the time stamps and the estimated relay positions. Owing to these properties, a monitoring delay does not occur in the proposed system, and the user accuracy is unaffected by the mobility of the airborne relays. Furthermore, unlike CAPS, the proposed algorithm is applicable even if the locations of the airborne relays and propagation times between the reference stations and airborne relays are not known in advance. However, the proposed system system may not properly operate at a location where the visibility of the airborne platforms cannot be guaranteed, similar to the airborne-based pseudolite system.

## 4. Simulation Results and Discussion

### 4.1. Simulation Assumptions and Construction

#### 4.1.1. Simulation Assumptions

In the proposed system, time synchronization between reference stations must be achieved for accurate position estimation. We assumed that reference stations are precisely synchronized with each other in the first and second simulations in order to compare the performance of the pseudolite-based and proposed systems in terms of the positioning algorithm, although some clock offsets could occur. However, we considered the offset of each reference station in the third simulation to show the feasibility of the proposed system.

In addition, we assumed that the position change of airborne relays, which results from the propagation time difference of navigation signals, during relay, is negligible. In the proposed system, the maximum difference between reference stations and airborne relays is about 130 km, because the distances between ground stations and airborne relays range from 20 km to 150 km. Thus, the propagation time of the navigation signal for the maximum range difference is about 0.4 ms, and the movement during that time is very small.

The pseudolite-based and proposed systems have the near-far problem when the distances from the pseudolites and airborne relays to the user are relatively different. In this study, we did not consider this issue because the problem can be resolved through existing schemes, such as pulsing techniques [1,7].

Finally, we assumed that the airborne-based pseudolite positioning system is asynchronous. Therefore, the reference transmitter and reference receiver were needed to provide double-differenced measurements for eliminating clock offsets. In the simulations, the time required to send the measurements of the reference receiver and ground receivers was regarded as negligible, because the measurements can be provided in real time using the data link.

#### 4.1.2. Simulation Model Construction

In the new positioning algorithm of the proposed system, the positions of airborne relays and users are sequentially calculated using relayed navigation signals. Because of the algorithm, monitoring delay is not incurred, unlike the airborne-based pseudolite systems, and thereby, the accuracy can be improved.

The accuracy in the regional navigation systems depends on the geometric position of segments. Furthermore, the proposed system and the airborne-based pseudolite system have similar structures, except for a reference transmitter and a reference receiver, which are required to eliminate the clock offsets in the asynchronous airborne-based pseudolite system. Therefore, we defined the same disposition of segments in order to be adequately comparable without the impact of geometric factors. The altitude of airborne relays and pseudolite was set at 20 km considering UAV and stratospheric airship altitudes. The user must receive at least four signals from the airborne relays or pseudolites to calculate its position. Therefore, the radius of the airborne relay and pseudolite trajectory was set to 90 km in order to ensure an overlap of each airborne platform coverage, because each platform coverage, which is the radius of the visible region on the ground with an elevation angle of 5°, is 195 km at an altitude of 20 km. We assumed that they are rotated along a circular orbit while maintaining a predetermined distance from each other. One of them is rotated relative to the center of a circle with a 10-km radius for good dilution of precision (DOP). The reference transmitter and the reference receiver for the airborne-based pseudolite system were set at the same position of (100, 100, 5000) to ensure the LOS with each ground receiver. Accordingly, we set the radius of the disposition of reference stations and ground receivers to 55 km with consideration of the coverage of the reference transmitter at an elevation angle of 5°. They were distributed in the same way as airborne platforms, but the locations were fixed.

**Figure 5:**
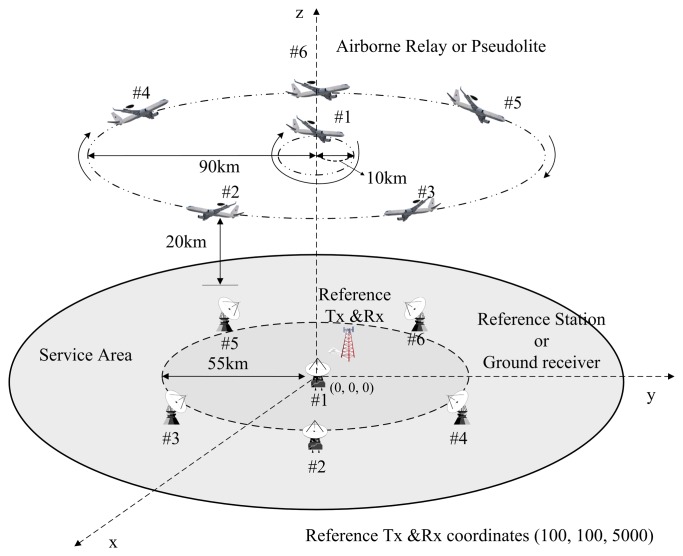
Segment disposition for simulations.

The control and master stations were not considered in the simulations because they do not influence positioning performance. Figure 5 shows the simulation configuration with six pseudolites and six ground receivers.

#### 4.1.3. Measurement Errors

The positioning systems discussed in this study are based on the code-phased pseudorange measurements at the meter level. There are several error sources that corrupt the measurements, such as the clock offset, multipath effect, atmospheric effects (e.g., tropospheric and ionospheric delays) and receiver noise. Herein, we consider three factors that contribute to airborne relay and user positioning measurement errors: the multipath effect, tropospheric delay and receiver noise. The ionospheric delay, which is the largest error in a pseudorange measurement, is not considered, because the airborne relays are located below the ionosphere.

The Hopfield model presented by Wang [15], which is stable for all ranges of elevation angles, is used to determine the tropospheric error. The tropospheric delay error consists of both dry and wet components; the error is defined as *m* = *d_dry_* + *d_wet_*. The expression is written as follows:

(8) $$d_{*}=\frac{10^{-6}}{5}\cdot N_{*}\cdot D\cdot \left({\left(1-\frac{h_{rx}}{h_{*}}\right)}^{5}-{\left(1-\frac{h_{tx}}{h_{*}}\right)}^{5}\right)\cdot \frac{h_{*}}{h_{rx}-h_{tx}},*\in \left\{\text{dry},\text{wet}\right\}$$

(9) $$\begin{array}{cc} {\text{N}}_{\text{dry}}=77.6\cdot \frac{P}{T}, & N_{\text{wet}}=22770\cdot \frac{f}{T^{2}}\cdot 10\frac{7.4475.\left(T-273\right)}{T-38.3} \end{array}$$

where N_*_ denotes the refractive index defined by Equation (9) *D* is the slope distance between the transmitter and receiver of the navigation signal, *h_rx_* and *h_tx_* respectively represent the receiver and transmitter heights, h_*_ is the fixed scaled height for the model (*h_dry_* = 42.7 km, *h_wet_* = 12 km) and *P*, *T* and *f* represent the atmospheric pressure, temperature and relative humidity, respectively (*P* = 1010.25 mbar, *T* = 291.15 K, *f* = 50%).

The multipath error is difficult to model, because the possible biases are most likely randomized in a kinematic environment. For simulations, the error due to multipath effects is provided using the SatNav toolbox considering GNSS-like signals. The SatNav toolbox forms zero-elevation angle equivalent pseudorange multipath errors by a linear autoregressive model, which is characterized by a standard deviation of approximately 1.6 m and a time constant of approximately 2 min. Before these errors are applied to pseudorange measurements, they are scaled by the factor $M\left(=1-\frac{{\text{tan}}^{-1}E}{{\text{tan}}^{-1}\left(\pi /2\right)}\right)$ where *E* is the elevation angle between the transmitter and receiver of the navigation signal [16]. In addition, the error due to receiver noise is generated by multiplying the standard deviation corresponding to the respective signal type by a normal distributed random number (*n* ∼ *N*(0,1)) [17,18]. The simulations of the airborne-based pseudolite system applied the same measurement error models described above.

### 4.2. Simulation Results and Discussion

To provide a basis for comparison, we performed simulations using MATLAB. First, the simulations with various numbers of segments were conducted to analyze the performance of the proposed system according to the geometry. Second, we compared the performances of the airborne-based pseudolite system and proposed system in terms of the accuracy corresponding to the delay and user distributions. Finally, to demonstrate the feasibility of the proposed positioning algorithm, we evaluated the performance of the proposed system if reference station clock offsets occurred.

#### 4.2.1. Relationship between Accuracy and Segment Geometry

As with the pseudolite-based positioning system, the accuracy of the proposed system depends on the geometry of the reference stations, airborne relays and the user. In the first simulation, we analyzed the performance while changing the geometry, depending on the number of segments and the user positions. The user error was calculated by averaging all errors at each position of the airborne relays, which moved at 50 m/s along the trajectory.

**Figure 6:**
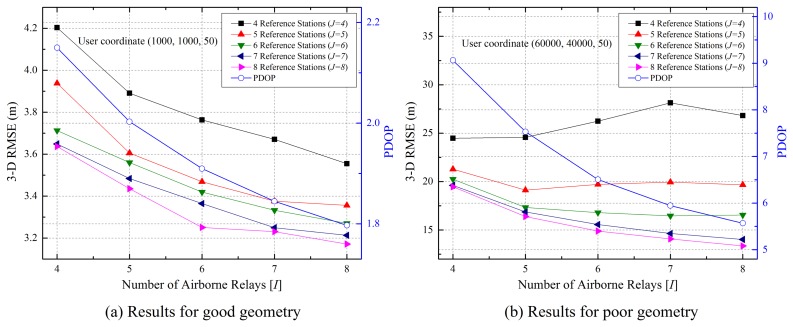
Performance with various combinations of segments.

Figure 6 shows the three-dimensional root mean square error (3D RMSE) of the user and the position DOP (PDOP) of the user with respect to airborne relays for good and poor geometries. The results of the good geometry show that user accuracy improves with an increase in the number of segments. On the other hand, the poor geometry results slightly differ when the number of reference stations is small. In the poor geometry, the deviation in the pseudorange errors between the airborne relays and user is large. This is due to the geometry of airborne relays with respect to the user. Therefore, the user error with the four airborne relays is more accurate because it is less affected by the deviation of pseudorange errors. However, the user accuracy is improved like the good geometry results as the number of segments increases, because the influence of geometry decreases. Based on these results, the remaining simulations were performed with six reference stations (*J* = 6) and four airborne relays (*I* = 4).

#### 4.2.2. Effect of Delay on User Accuracy

In this section, we describe the simulations we conducted to analyze the effect on user accuracy in accordance with the monitoring delay and to compare the airborne-based pseudolite system and proposed system performances. For a fair simulation, the same segment disposition and measurement models were used. In addition, the position estimation of the user was conducted without interpolation/extrapolation for mitigating errors.

**Figure 7:**
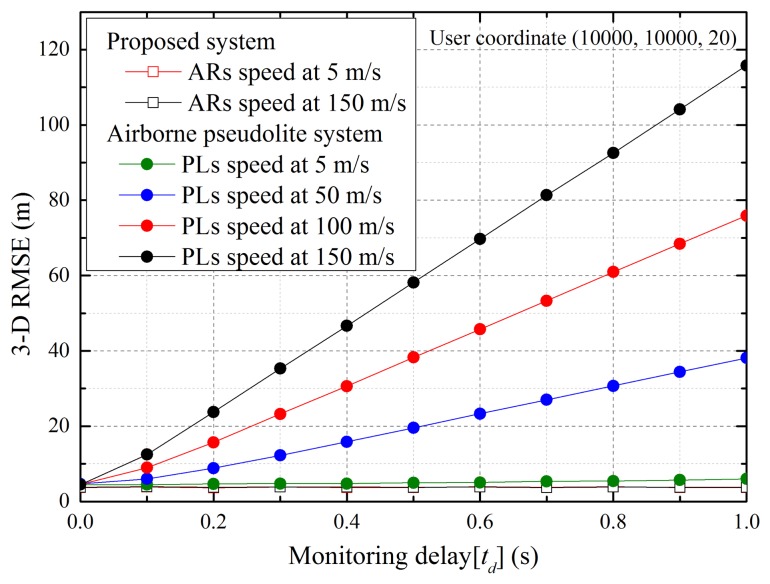
Variation in accuracy according to monitoring delay.

Monitoring delay is caused by the generation and propagation times of navigation signals, the processing and calculation times of the control station and other factors. This delay is difficult to accurately predict because it can vary according to system specifications and external factors. Therefore, we analyzed the performance while varying the delay and speed of pseudolite platforms and airborne relays. Figure 7 illustrates the 3D RMS errors of the user located at (10,000, 10,000, 20) according to the monitoring delay and speed of the pseudolites and airborne relays. The results show that user accuracy decreases as the monitoring delay and platform speed increase. Although the inaccuracies caused by the monitoring delay can be mitigated if the pseudolites are mounted on low-speed platforms, the delay remains a problem of the airborne-based pseudolite positioning system. On the other hand, in the proposed system, the monitoring delay does not occur, and the speed of the airborne relays does not affect performance, because the positions of the airborne relays and user are sequentially estimated by the user using relayed navigation signals.

**Figure 8:**
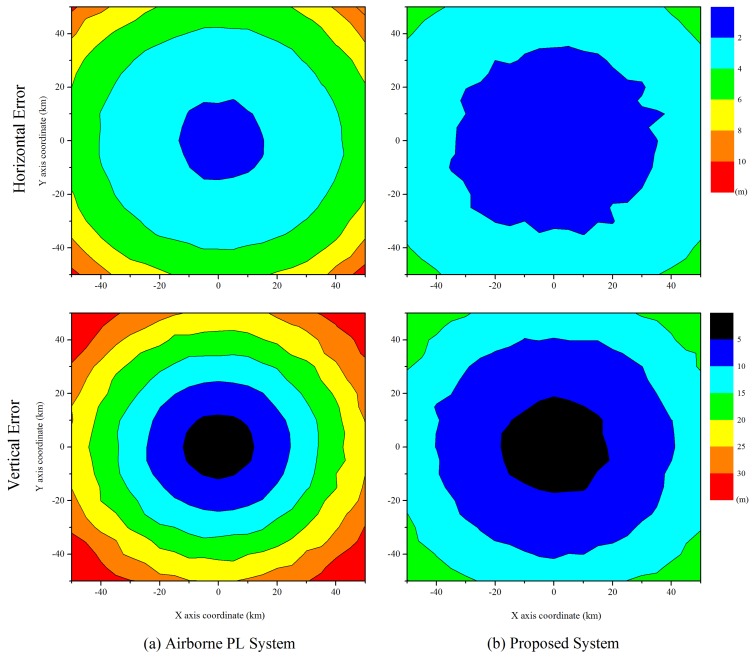
Horizontal and vertical accuracies of the user in a service area.

Figure 8 illustrates the horizontal and vertical error distributions of the user in a service area of 100 km × 100 km,, and the color represents the user error. In the simulations, we set the monitoring delay to 0.1 s and the platform speed to 50 m/s. The simulation results show that the vertical error is larger than the horizontal error, and the accuracy at the center is more precise. These results are due to the positioning technique and geometry between the airborne relays and user. Because all airborne relays and pseudolites are located at an altitude of 20 km in the same direction, the major axis of the error ellipse is in the vertical direction. The center exhibits better accuracy than other areas, because it can usually guarantee large elevation angles. In addition, we observe that the proposed system is more accurate than the airborne-based pseudolite system over a wide area. In the proposed system, airborne relays transfer all navigation signals to the user, and the pseudorange measurements between the airborne relays and user are estimated by averaging all measurements for each airborne relay. Therefore, it achieves more precise results, because the pseudorange measurement error is reduced.

#### 4.2.3. Performance Considering Reference Station Clock Offsets

Time synchronization between reference stations in the proposed system should be maintained for precise position estimation. However, because it is difficult to achieve perfect synchronization in a practical system, the performance of the proposed system may be degraded. In this simulation, the proposed system was evaluated for cases in which clock offsets occur. According to [4], the Locata system achieves nanosecond accuracy through a wireless synchronization method called TimeLoc. Using that as a reference, the clock offsets in nanoseconds were randomly generated based on uniform distribution (*b_rj_* ∼ *U*(0, *N*)), and we evaluated the performance of the proposed system while varying *N*. The simulation settings and user positions were set to be equal to those of the first simulation.

**Figure 9:**
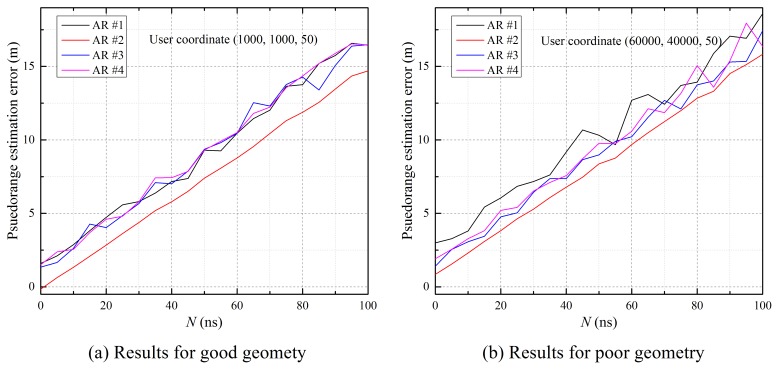
Error of estimated pseudorange measurements.

Table 1 shows the RMS error budgets for simulation, which are generated by error models according to user position. As described in Section 3.2, the pseudorange measurements between each airborne relay and user can be estimated using Equation (5). Figure 9 illustrates the difference between the actual distance and the estimated pseudorange, which includes residual errors, such as tropospheric delay, multipath delay and noise. The results show that the pseudorange errors tend to increase when *N* increases, and the error for poor geometry is large due to residual errors according to geometry. In addition, the error of pseudorange measurements between airborne Relay 2 and the user is lower than the rest, because airborne Relay 2 is rotated relative to the center of distribution, which guarantees a large elevation angle and good DOP with reference stations. These measurement errors affect the user estimation result.

**Figure 10:**
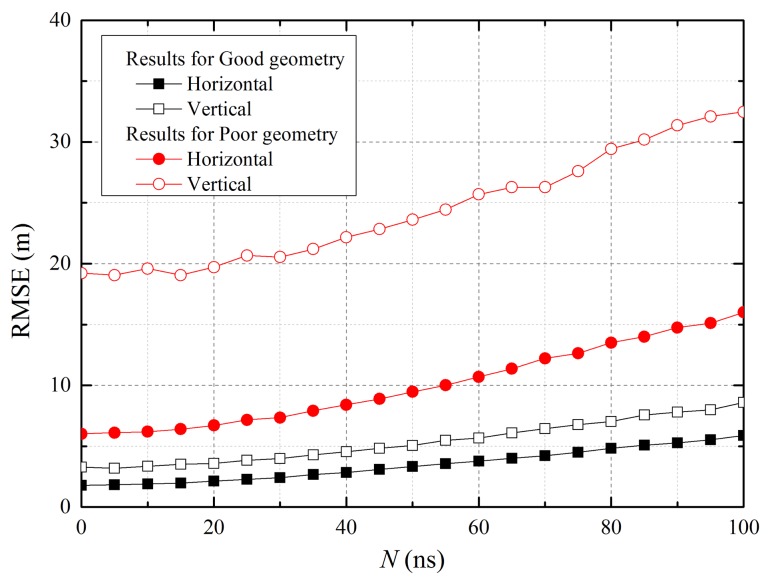
RMS error of the user according to clock offset.

Figure 10 shows the horizontal and vertical RMS errors of the user according to the clock offset. It is not surprising that the error for the poor geometry position is greater than that of the good geometry because the residual errors are large with a low elevation angle, as shown in Table 1. Moreover, the errors become worse as the offset increases. However, if the time synchronization among the reference stations is maintained within 20 ns, the errors slightly increase. This result demonstrates that the proposed system can operate regardless of the existence of clock offsets in the reference stations.

## 5. Conclusions

In the airborne-based pseudolite positioning system, the delay from monitoring pseudolite positions causes performance degradation, whereas the problems caused by geometrical factors are not as significant as with ground-based systems. To address these problems and develop an independent positioning system, we have proposed an airborne relay-based positioning system and algorithm. In the proposed system, the positions of airborne relays are first determined by the user using the TDOAs of relayed signals, and then, the user position is calculated based on the estimated relay positions.

To evaluate the proposed system, various simulations were performed using MATLAB. Based on the simulation results, we have confirmed that the delay due to monitoring and the effect of airborne relay mobility do not occur in the proposed system because the position estimation of the airborne relays and user is simultaneously performed at the user terminal. Moreover, the proposed system ensures a higher accuracy over a wide area than the airborne-based pseudolite system. Finally, intentional clock offsets between the reference stations were generated to verify the feasibility of the proposed system with consideration of a practical system. The results have shown that the proposed system still guarantees high accuracy if the time synchronization is maintained within tens of nanoseconds.

## Figures and Tables

**Table 1:** Error budget for the simulation.

**Error Source**	**Good Geometry**	**Poor Geometry**
Tropospheric Delay	1.48 (m)	1.99 (m)
Multipath	1.08 (m)	1.17 (m)
Receiver Noise	0.25 (m)	0.25 (m)

## Appendix

### A. Derivation of Navigation Equations for an Airborne-Based Pseudolite System

In this section, we reformulate the navigation equations of the airborne-based pseudolite positioning system, which is based on the IGPS method, in order to have an appropriate comparison. Because we assume that the system is asynchronous, double-difference processing is used in both pseudolite and user position estimations to eliminate clock offsets.

For position estimation of pseudolites, the measurements of the pseudolites and reference transmitter at each ground receiver are differenced as ∇pltρgk=ρgkpl−ρgkt where $\rho_{g_{k}}^{p_{l}}$ and $\rho_{g_{k}}^{t}$ signify the pseudoranges from the *l*-th pseudolite and reference transmitter to the *k*-th ground receiver. Using these single-differenced measurements, the double-differenced measurement for the *l*-th pseudolite/reference transmitter and the first/*k*-th ground receivers can be written as follows [9]:

(A1) ∇pltρgkΔg1ρ=egkpl⋅(Rpl−Rgk)−egkt⋅(Rt−Rgk)−eg1pl⋅(Rpl−Rg1)+eg1t⋅(Rt−Rg1)+∇pltgkΔg1τ+∇pltgkΔg1m+∇pltgkΔg1n,1≤k≤K,1≤l≤L

where ∇pltρgkΔg1ρ=∇pltρgk−∇pltρg1, $e_{g_{k}}^{p_{l}}$ and $e_{g_{k}}^{t}$ indicate the unit vectors of the *k*-th ground receiver to the *l*-th pseudolite and reference transmitter and ***R****^pl^*, ***R****_gk_* and ***R****_t_* denote the position vectors of the *l*-th pseudolite, *k*-th ground receiver and reference transmitter, respectively.

With the double-differenced measurement containing no pseudolite and ground receiver offsets, the navigation equation, which estimates the *l*-th pseudolite position, can be constructed as follows:

(A2) [eg2pl−eg1pleg3pl−eg1pl⋮egKpl−eg1pl]⋅Rpl=[pg2g1pg3g1⋮pgKg1]−[∇pltg2Δg1τ+∇pltg2Δg1m+∇pltg2Δg1n∇pltg3Δg1τ+∇pltg3Δg1m+∇pltg3Δg1n⋮∇pltgkΔg1τ+∇pltgkΔg1m+∇pltgkΔg1n]

where pgkg1=∇tgkplΔg1ρ+egkpl⋅Rgk+egkt⋅(Rt−Rgk)−eg1pl⋅Rg1−egkt⋅(Rt−Rg1).

As explained in Section 2, a delay due to the monitoring of pseudolite positions occurs. Owing to this factor, the user position accuracy is affected, because the pseudolites generate navigation signals based on their estimated positions after moving for as long as the delay. Considering this delay, the single-differenced measurements of the user and reference receiver between the first and *l*-th pseudolites can be written as follows [19]:

(A3) ∇p˜lp˜lρu=eupl⋅(Rpl−Ru)−eup1⋅(Rp1−Ru)−(bp˜l+bp˜1)+∇p˜lp˜1τu+∇p˜lp˜1mu+∇p˜lp˜1nu∇p˜lp˜1ρr=eupl⋅(Rpl−Rr)−erp1⋅(Rp1−Rr)−(bp˜l+bp˜1)+∇p˜lp˜1τr+∇p˜lp˜1mr+∇p˜lp˜1nr

where $e_{u}^{p_{l}}$ and $e_{r}^{p_{l}}$ respectively denote the unit vectors of the user and reference receiver to the *l*-th pseudolite, ***R****_u_* and ***R****_r_* are the position vectors of the user and reference receiver, respectively, and *b^p̃l^* is the *l*-th pseudolite clock offset. In Equation (A3), *p̃_l_* represents the *l*-th pseudolite, which moved from the estimated position during the delay of *t_d_* due to monitoring. We define the position vector of the moved pseudolite as *R_p̃l_* = *R_pl_* + Δ*R_pl_* with the position displacement of the pseudolite as Δ*R_pl_* = *V_pl_* × *t_d_*, where *V_pl_* is the velocity vector of the *l*-th pseudolite. Using the single-differenced measurements, the double-differenced measurement containing no pseudolite and user clock offsets can be constructed as:

(A4) (eup1−eupl)⋅Ru=∇p˜lp˜1uΔrρ−eupl⋅Rpl+erpl⋅(Rpl−Rr)+eup1⋅Rp1−erp1⋅(Rp1−Rr)−∇p˜lp˜1uΔrτ−∇p˜lp˜1uΔrm−∇p˜lp˜1uΔrn≡qp˜lp˜1−∇p˜lp˜1uΔrτ−∇p˜lp˜1uΔrm−∇p˜lp˜1uΔrn.

From Equation (A4), the navigation equation for user positioning given *L* pseudolites is derived as Equation (A5). Finally, the user position is determined by the nonlinear least squares method.

(A5) [eup1−eup2eup1−eup3⋮eup1−eupL]⋅Ru=[qp˜2p˜1qp˜3p˜1⋮qp˜Lp˜1]−[∇p˜2p˜1uΔrτ+∇p˜2p˜1uΔrm+∇p˜2p˜1uΔrn∇p˜3p˜1uΔrτ+∇p˜3p˜1uΔrm+∇p˜3p˜1uΔrn⋮∇p˜Lp˜1uΔrτ+∇p˜Lp˜1uΔrm+∇p˜Lp˜1uΔrn]
